# Trust Dynamics and Equity in Public Health in Canada: Protocol for a Mixed Methods Project in the Postpandemic Era

**DOI:** 10.2196/75199

**Published:** 2025-11-28

**Authors:** Nazeem Muhajarine, Cory Neudorf, Khatira Mehdiyeva, Fionnuala Braun, Syed Jafar Raza Rizvi, Zili Zhou, Thilina Bandara, Katrina Baudelaire, Sohana Sadique, Daniel A Adeyinka, Sahana Ramamoorthy, Ninan Abraham, Kimberly Huyser, Claire Betker, Mary Jessome, Tamara Chavez, Kim L Lavoie, Ève Dubé

**Affiliations:** 1 Department of Community Health and Epidemiology and Saskatchewan Population Health and Evaluation Research Unit College of Medicine University of Saskatchewan Saskatoon, SK Canada; 2 Department of History Carleton University Ottawa, ON Canada; 3 College of Medicine University of Saskatchewan Saskatoon, SK Canada; 4 School of Public Health University of Saskatchewan Saskatoon, SK Canada; 5 Department of Zoology Department of Microbiology and Immunology University of British Columbia Vancouver Canada; 6 Department of Sociology University of British Columbia Vancouver, BC Canada; 7 National Collaborating Centre for Determinants of Health (NCCDH) St. Francis Xavier University Antigonish, NS Canada; 8 Montreal Behavioural Medicine Centre Department of Psychology Université du Québec à Montréal (UQAM) Montreal, QC Canada; 9 Department of Anthropology Université Laval Montreal, QC Canada

**Keywords:** COVID-19 pandemic, trust dynamics, Canada, mixed methods, health equity, public health

## Abstract

**Background:**

The COVID-19 pandemic impacted trust in public health, medical care, scientific, and governmental institutions; this influenced health, health-seeking behaviors, and adherence to public health measures. Understanding how trust evolves is necessary for informing future public health crisis management and strategies. Rebuilding public trust is key to pandemic preparedness worldwide. This research protocol examines these trust dynamics, their determinants, and their implications.

**Objective:**

This study aimed to investigate the evolution of public trust in scientists, public health and medical care institutions, governments, and social and personal networks during the COVID-19 pandemic. It sought to identify key factors contributing to trust maintenance or erosion, particularly in marginalized and minority communities, and understand the impact of trust level on public health adherence.

**Methods:**

A sequential, explanatory mixed methods approach was implemented, consisting of an initial quantitative survey followed by qualitative interviews. The nationwide representative survey included 5607 Canadian residents as of May 2024. The questionnaire assessed trust in 6 key trust sources before and during the pandemic: provincial and federal governments, public health authorities, medical care providers, health scientists, and social and personal networks. To complement, 41 qualitative interviews were conducted to understand participants’ lived experiences, perceptions, and how trust played into both. Data have been analyzed using quantitative statistical techniques and qualitative phenomenological analysis, and the results have been integrated to derive comprehensive insights. All phases of data collection and analysis were finalized by early 2025. The project then advanced to paper preparation, dissemination at national conferences, and knowledge-translation activities, including the report development and public-facing outputs, scheduled for completion in end-2025.

**Results:**

This Canadian survey included participants from all 10 provinces and 2 territories; the provinces and territories’ samples matched the proportions of each in the overall Canadian population. Age and sex or gender were well distributed across the sample. Additionally, 18.6% (1040/5607) identified as an ethnic minority, 12.7% (710/5607) identified as Indigenous, including 7.2% (403/5607) First Nations, 4.8% (270/5607) Métis, and 0.4% (25/5607) Inuit. Fifty-five percent (3094/5607, 55.2%) had received at least 1 dose and planned to stay up to date with booster recommendations, while 36.6% (2052/5607) were vaccinated but did not intend to receive additional doses. A small percentage, 0.7% (39/5607), had not yet been vaccinated but were open to it, whereas 6.4% (359/5607) had not received a vaccine and did not plan to take a vaccine.

**Conclusions:**

The COVID-19 pandemic has underscored the critical role of trust in public health behavior and crisis response. This study explores how consistent, transparent communication and equity-driven policies may contribute to maintaining public trust, particularly among marginalized communities. By examining trust dynamics and identifying potential disparities, this study aims to inform evidence-based public health strategies and improve preparedness for future health emergencies.

**International Registered Report Identifier (IRRID):**

DERR1-10.2196/75199

## Introduction

### Background

March 11, 2025, marks the five-year anniversary of the official declaration of the COVID-19 pandemic by the World Health Organization, a milestone that reflects the immense challenges faced and the ongoing impacts of this global health emergency. The COVID-19 pandemic, one of the most devastating public health crises in modern history, emerged at a time when the world, including Canada, was largely unprepared. Canada’s first confirmed case was reported in Ontario on January 25, 2020, and within weeks, the country recorded its first death from the virus on March 09, 2020. On March 11, 2020, the World Health Organization declared the outbreak a pandemic [1]. In response, Quebec declared a state of public health emergency on March 13, followed by all other provinces and territories by March 27, as the virus spread rapidly across the country [2]. By December 2020, the vaccines had been developed and brought to people, and the rollout began across many countries, including in Canada, marking a critical and definitive turning point in the fight against the virus.

### Government Response and Canada in the Global Context

In the early stages of the pandemic, Canada prioritized restricting international travel, based on the belief that limiting travel from the virus’s point of origin—Wuhan, China—could help reduce its introduction into the country [3]. Early measures included travel advisories, closure of the Canada-US border, and a mandatory 14-day self-isolation for those entering the country [4]. As COVID-19 transitioned from being a concern primarily for travelers to widespread community transmission, additional public health measures were implemented, including social distancing, hand hygiene, mask mandates, and the closure of schools and daycares [5]. Many workers transitioned to remote work, public gatherings were prohibited, restaurant capacities were limited, and public spaces such as gyms and recreational facilities were closed. While the federal government provided oversight relating to national measures, the pandemic countermeasures closest to people were introduced and enforced by provincial and territorial governments, depending on local conditions [2].

Worldwide, Canada’s response in the first 2 years of the pandemic outperformed many other Group of Ten (G10) countries [6]. Canada had the second-lowest infection and mortality rate among the G10, just behind Japan [7]. According to the Oxford Stringency Index, which measures the intensity of governmental responses based on indicators such as travel bans, school closures, and workplace lockdowns, Canada ranked as the second-most stringent country among the G10 during the first 2 years of the pandemic, just behind Italy [4]. Unlike many other nations, Canada’s response was characterized by its prolonged and sustained measures, lasting more than 2 years.

### Overall Impact of the COVID-19 Pandemic in Canada

The impact of the COVID-19 pandemic on Canadians was profound, affecting nearly every aspect of daily life. Beyond physical health, mental health became a major concern. Canadians experienced heightened anxiety, depression, and stress due to isolation, uncertainty, and economic struggles [8,9]. Alongside psychological distress, alcohol and substance use spiked [8,10], while studies also reported rising rates of self-harm and suicidal ideation compared to prepandemic levels [11-13]. Vulnerable populations—such as children and youth, low-income groups, racial and ethnic minorities, and immigrants—were disproportionately affected, facing even greater mental health challenges [14-18]. The economic impact was also severe, with rising unemployment, government debt, and inflation [7]. Domestic violence and relationship conflicts also increased, particularly among those who experienced job loss or reduction [19]. These economic hardships deepened the existing social inequities, with marginalized communities bearing the brunt of the effects [20,21].

### Change in Trust During the Pandemic

Although Canada’s COVID-19 outcomes in terms of infection rates and death rates were relatively moderate compared to many countries, especially the United States, it still faced criticism for its pandemic management when compared to exemplars such as Australia, New Zealand, and South Korea [22].

A key point of contention was the vaccine mandate, which required proof of vaccination for discretionary activities such as dining in restaurants, entering gyms, and cross-border travel. This policy sparked widespread protests, including the “freedom convoy” in early 2022 [23].

Canada initially saw low vaccine uptake, due to supply issues, but once vaccines were acquired in plentiful numbers, Canada’s vaccination rates climbed rapidly [7,24]. The vaccine uptake, however, plateaued at a high level, due to growing vaccine hesitancy and refusal, particularly for booster doses in 2021 and 2022 [25,26]. A major contributing factor was declining trust in governments and in public health agencies, which negatively influenced the public acceptability of COVID-19 vaccines [27,28].

The rapid development of COVID-19 vaccines, completed in just 11 months, raised skepticism, as vaccine development typically takes years. In the face of rapidly developing and changing scientific data, trust in government and public health agencies becomes crucial for vaccine uptake [29]. One study showed that public trust in health care providers, public health agencies, and pharmaceutical companies significantly shaped their perception of vaccine effectiveness and safety, and, ultimately, their willingness to get vaccinated [27]. A systematic review and meta-analysis conducted in Canada revealed that in 2021, a total of 1 in 5 people did not want to get vaccinated and that vaccine hesitancy and unwillingness were especially higher among women, rural residents, non-White individuals, and those with lower educational levels [25].

Another key factor that contributed to the erosion of trust was the rapid policy reversals and inconsistent messaging [30]. For instance, the Public Health Agency of Canada initially advised against mask-wearing for asymptomatic individuals, only to later mandate masks in public spaces. A survey in Ontario revealed that more than half of the participants had encountered COVID-19 misinformation and had challenges identifying or appraising COVID-19 information [31]. While provincial leaders, especially in British Columbia and Quebec, were praised for their effective communication, the federal government, as were a few other provincial governments, struggled to provide unified, transparent messaging. The reliance on vague objectives such as “flattening the curve” was widely criticized for lacking clarity and concrete targets, making it harder for the public to rally behind a shared goal. In contrast, countries such as New Zealand and Australia, with clearer and more ambitious targets such as “virus elimination,” were more successful in maintaining public support [22].

The 2021 Edelman Trust Barometer revealed that public trust in the Canadian government dropped by 11% between May 2020 and January 2021, alongside a decline in trust in traditional media, social media, and search engines [32]. Additionally, the pandemic and the related restrictions imposed impacted vulnerable communities disproportionately, exacerbating the existing inequalities, which added another layer of complexity to public trust [21].

As of 2025, Canada has largely entered a post-pandemic phase, though COVID-19 continues to circulate. The pandemic not only exposed vulnerabilities in Canada’s response but also raised concerns about the government’s ability to manage future emergencies effectively. The pandemic’s long-term effects, including the erosion of public trust, remain a significant and immediate challenge. Understanding this shift in trust and rebuilding trustworthiness and trust are crucial for Canada’s preparedness for future crises.

### Trust Dynamics and Equity in Public Health Project

Trust can be conceptualized as a combination of behavioral practices and norms, perceptions and preferences, as well as the functional and institutional properties of interactions between individuals and organizations [33-35]. This conceptual framing informs the types of interventions that may be designed to rebuild or strengthen trust and trustworthiness. The Trust Dynamics and Equity in Public Health Project, a program of interlinked studies, investigates the evolution of public trust in scientists, public health, medical care, and governmental institutions during the COVID-19 pandemic. It sought to understand the extent to which Canadians trusted their own social and personal networks (traditional media, social media, and friends, family, and faith leaders) as sources of information during the pandemic. This research aims to understand how trust was maintained or eroded, particularly among marginalized and minority communities, and how this trust influenced adherence to public health measures and health care–seeking behaviors. Key objectives include (1) investigating the evolution of public trust in scientists, public health authorities, medical care providers, and governments during the pandemic; (2) identifying factors influencing trust, specifically the erosion of trust or maintenance of trust among Canadians at large and in equity-seeking communities; (3) identifying factors that are associated with a change in trust (trust increased, decreased, or stayed the same) in May 2024 compared to before the pandemic (before March 2020); and (4) understanding how Canadians describe trust and trust change during the pandemic, how it impacted vaccine receipt, and how to rebuild trust moving forward.

**Figure 1:**
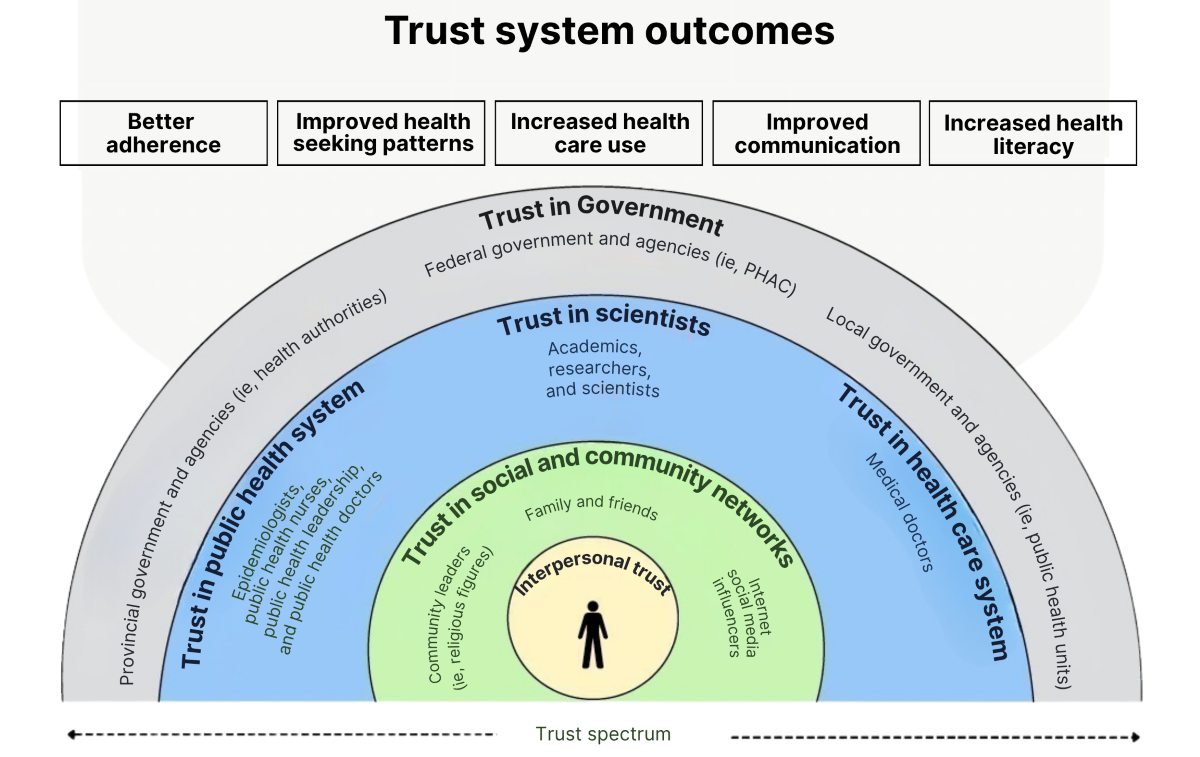
Concept framework of Trust Dynamics and Equity in Public Health Project. PHAC: Public Health Agency of Canada.

This study is guided by a concept map (Figure 1) developed through a literature review. This concept map serves as the framework for understanding trust dynamics and their impact on health behaviors. The concept map highlights the multilayered nature of trust in government, scientists, the health care system, public health, social and community networks, and interpersonal trust. It illustrates how different sources of trust interact and influence health behaviors and crisis responses. Trust in these systems is essential for public compliance, effective communication, and improved health care outcomes. The survey questionnaire was designed and structured based on the concepts in the map.

## Methods

### Study Setting and Design

This study enrolled participants from across Canada, all 10 provinces, and 2 of the 3 territories (a sample from Nunavut was not drawn), capturing a diverse and representative sample of the adult population. The sequential mixed methods approach, quantitative followed by qualitative (sequential quantitative → qualitative), was chosen for this research to provide both breadth and depth in understanding trust dynamics in public health. In the first phase, the national survey of Canadian adults allowed for the collection of large amounts of data, identifying trends and patterns in trust perceptions across diverse demographic groups and places. However, trust is a complex and context-dependent phenomenon that cannot be fully captured through survey responses alone. By incorporating qualitative interviews in the second phase, this study gained deeper insights into the motivations, experiences, and concerns underlying the observed quantitative trends. This methodological combination ensured a comprehensive analysis, strengthening the validity and applicability of the findings for public health policies and interventions.

The recruitment phases have been completed, with a population representative sample of 5607 Canadian adult residents surveyed to assess trust perceptions and attitudes and behaviors related to COVID-19 vaccines. The sample size was informed by precision-based considerations: assuming a conservative prevalence of 50% (where variance is maximal), approximately 2400 respondents would be required to achieve ±2% precision at the 95% confidence level. Our achieved sample of 5607 more than doubles this requirement, yielding national prevalence estimates with a margin of error below ±1.5%. This larger sample also ensured sufficient statistical power (>80%) to detect meaningful subgroup differences—on the order of 5-7 percentage points—across sociodemographic groups (eg, Indigenous vs non-Indigenous respondents, age cohorts, and regions), and to examine changes in trust before and during the pandemic with high precision.

Additionally, 41 in-depth qualitative interviews have been conducted to explore underlying motivations and concerns.

### Population Survey

The quantitative phase of this study involved an online survey designed and developed by the University of Saskatchewan team in collaboration with Leger, the largest Canadian-owned research and analytics company. The questionnaire comprised approximately 70 questions, including screening and demographic questions (Multimedia Appendix 1). Three attention check questions were inserted into the survey, approximately every 4-5 screens. Specifically, these directed questions asked participants to give specific answers (eg, “This is a control question. Mark ‘agree’ and ‘move on’”). The survey was translated and programmed in both English and French by Leger, ensuring accessibility for respondents in their preferred language. Respondents were primarily recruited through the LEO (Leger Opinion) panel, Canada’s largest consumer panel with nearly 400,000 active members. For respondents in the Northwest Territories and Yukon, recruitment was conducted through the Hard-to-Reach solution by Leger DGTL (digital), which leverages digital marketing strategies to engage niche populations. A stratified random sampling method was used to ensure representative coverage. The survey targeted Canadian residents aged 18 years and older who had resided in Canada for most or all of the COVID-19 pandemic. Additional quotas were implemented to ensure adequate representation of Indigenous Canadians, young adults (18-34 years), and residents of the Northwest Territories and Yukon.

The online survey was conducted between May 1 and May 29, 2024. Respondents received personalized invitation emails containing a unique URL, allowing them to complete the survey at their convenience using a computer, smartphone, or tablet. The average survey completion time was 13 minutes and 23 seconds. Before full deployment, a pretest was conducted on May 1, 2024, with 44 respondents to assess completion time, question clarity, and logical structure. No major revisions were required following the pretest, and the data collected were included in the final dataset. To prevent duplicate entries, LEO assigned unique IDs to each respondent, ensuring that once a survey was completed or terminated, re-entry was not possible. The final sample consisted of 5607 fully completed responses from an initial pool of 7858 invitees. Of these, 710 respondents identified as Indigenous Canadians (“First Nations”: n=403, “Métis”: n=270, “Inuit”: n=25, “Prefer to self-identify”: n=11, and “Don’t know/Refused”: n=1). A 2-step approach was implemented to achieve a representative sample: (1) Recruitment and sampling were monitored and adjusted in real time based on the latest Canadian Census data (2021) to ensure demographic balance, and (2) at the conclusion of data collection, weighting adjustments were applied to align the final dataset with population benchmarks for age, sex, and region.

**Figure 2:**
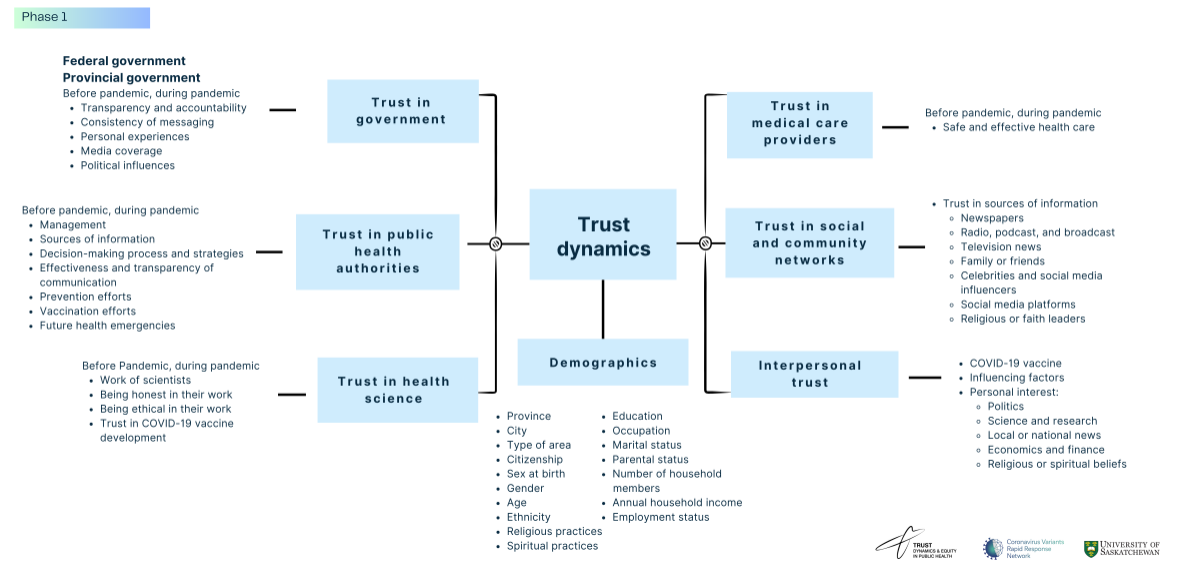
Questionnaire content: public trust before and during the COVID-19 pandemic.

The questionnaire was designed to assess 6 primary sources of trust, with participants reflecting on their trust levels before and during the COVID-19 pandemic, alongside comprehensive demographic data, as described in Figure 2. Respondents provided insights into their trust in provincial or territorial government, federal government, public health authorities, medical care providers, health scientists, and social and personal networks. These trust dimensions were explored in relation to transparency, communication strategies, decision-making processes, and sources of information. Additionally, demographic questions covered aspects such as age, sex, gender, ethnicity, born in or outside of Canada, education, occupation, parental status, household income, and adherence to spiritual beliefs and practices to provide a detailed understanding of trust variations across different population groups.

### Statistical Methods

The survey data were analyzed using descriptive and inferential statistical techniques in R (The R Foundation for Statistical Computing) and Stata (StataCorp LLC). Descriptive statistics summarized demographic characteristics such as age, gender, ethnicity, education, occupation, household income, adherence to spiritual beliefs and practices, and other socioeconomic factors; geographic factors such as province or territory of residency, rural or urban residence; vaccine related attitudes and behaviors such as trust in the vaccine, willingness to receive the vaccine; and sources of trust such as provincial or territorial government, public health authority, health scientists, and medical care providers. Bivariate analyses using cross-tabulations with chi-square tests examined relationships between trust and trust changes of 6 primary sources of trust and demographic and socioeconomic, geographic, and vaccine-related variables. Multinomial logistic regression then identified factors associated with trust (ie, trust, distrust, preferred not to answer, or neutral), trust change categories (trust increased, decreased, no change, or preferred not to answer), adjusting for demographic and economic factors, geographic and vaccine-related factors. Analysis was conducted using a survey-weighted sample to represent the population benchmarks for age, sex, and region, and statistical significance was set at a 5% level.

### In-Depth Interviews

Participants were recruited after completing a quantitative survey. At the end of the survey, participants were allowed to indicate interest in a follow-up qualitative interview. The rationale for this recruitment strategy was both practical (access to participants) and by design, to gain further insight into answers given in the survey, though participants’ initial survey answers and consequent interview responses were not linkable. The quantitative survey that we first conducted provided a broad set of information for understanding how Canadians experienced trust in public health actors, governments, and their communities. However, we recognized that this quantitative analysis, while providing an overview, required a deeper reading to gain insights into individual experiences of trust during the pandemic. Furthermore, some participants chose the “preferred not to answer” response alternative for certain questions on the quantitative survey, particularly questions dealing with spiritual beliefs, gender, and ethnicity (n=285, n=74, and n=99, respectively). This suggested that a deeper investigation was needed to understand the complex dynamics of public trust during the pandemic. We developed a qualitative interview, both expanding on and responding to information gleaned from the quantitative results. This interview guide was internally reviewed for dependability, and the process and changes made were documented. The main questions we framed the qualitative protocol on were (1) What did participants remember most strongly about the COVID-19 pandemic? (2) How did participants define what trust means to them? (3) How did participants feel about public health interventions from scientists and policy makers during the pandemic? (4) Did participants’ trust in government information, health authorities, community leaders, and family change over the course of the pandemic? If so, how? (5) What actions would participants suggest policy makers and public health actors take in future pandemics to increase public trust?

Before beginning the interview process, all members of the interview team spent significant time discussing the volatility of our subject matter. Aware that discussing the pandemic, trust, and vaccination could compound trauma for participants, we were mindful of interviewer and participant safety throughout the interview process. All team members completed the Tri-Council Policy Statement: Ethical Conduct for Research Involving Humans certification. The team also undertook a day-long training session on trauma-informed interview practices and was diligent in recognizing their own positionality and bias, as well as preparing themselves to respond to participants’ potential trauma [36]. Some concerns were raised regarding interviewee safety, particularly if interviewees chose to keep their cameras on during Zoom (Zoom Communications, Inc) interviews. However, after consulting with the whole interview team, a consensus was reached that, in a project dealing with trust, it was deemed that, as researchers, it was our responsibility to set a base of trust by sharing our names and faces with interviewees.

Before the interview, participants were given a list of questions to consider that were thematically related to, but not direct copies of, the interview questions (Multimedia Appendix 2). Interviews lasted between 20-60 minutes and were conducted either over the phone or via Zoom, according to the participant’s preference.

We used a descriptive phenomenological approach in our qualitative interviews, using the frame of trust to understand participants’ lived experiences of the pandemic, and how these lived experiences in turn allowed them to make meaning of what “trust” meant during the pandemic, and as it has receded. This approach required the deep analysis of participants’ lived experiences, allowing us to investigate their “lifeworld” [37]. As Dahlberg [37] describes, the “lifeworld” is the “essence of that which is already there”—that is, the everyday experiences, realities, and events that underpin how we make sense of our lives. In our interviews, we used the phenomenon of public trust during the COVID-19 pandemic and sought to understand the meaning participants derived from this phenomenon, based on their descriptions of their “lifeworld” [38]. This approach is particularly appropriate to understanding trust, being defined as an experience that is unique to the lived experiences and consequent decisions of the trustor, and how these in turn affect the trustor’s relationship with the trustee [35,39]. The phenomenological approach also required the research team to focus fully on participants’ perceptions, descriptions, and reflections, and avoid interpreting these results through their own meaning-making frames. To facilitate credibility at all stages of this process, interviews were in-depth, and participants were invited to lead the conversation on topics they felt were important. This contextual richness also allows for transferability—the reader of this study will be able to fully interpret the relevance of the data in further understanding public trust in Canada, through the detailed descriptions provided by the participants, and included in knowledge dissemination activities. Further, team members met frequently to discuss how they made meaning of interview responses, to ensure they were mindful of preconceived biases.

Analysis began during the frequent meetings held by the research team, where emerging themes and patterns were discussed. After the interviews were complete, transcripts were uploaded to NVivo 14 (Lumivero). Adhering to the phenomenological approach, researchers once again “bracketed” their own preconceptions during coding, focusing on themes that emerged about participants’ lived experiences as they were described in the interviews [40]. An initial codebook was created based on consensus, and new codes were only added after a meeting where consensus was reached. To ensure confirmability, after the initial coding was completed by 2 team members, a third team member also coded a subset of the interviews a second time, validating the initial codes.

### Mixed Methods Approach

To integrate and interpret the 2 datasets, a sequential-explanatory mixed methods approach was applied. This approach required that the quantitative phase be completed first, followed by the qualitative phase, allowing the qualitative phase to expand on findings from the quantitative survey [40,41]. The integration of the quantitative and qualitative data was guided by the thread approach, which requires that researchers review both components of the study at the analysis phase to identify key themes and questions requiring further investigation [42]. These key themes or “threads” are then “followed” across the study [43]. As the threads are followed, more depth and nuance are added to the initial theme, meaning that the research team was able to glean a deeper understanding of these themes by interpreting them across both datasets, instead of in isolation.

**Figure 3:**
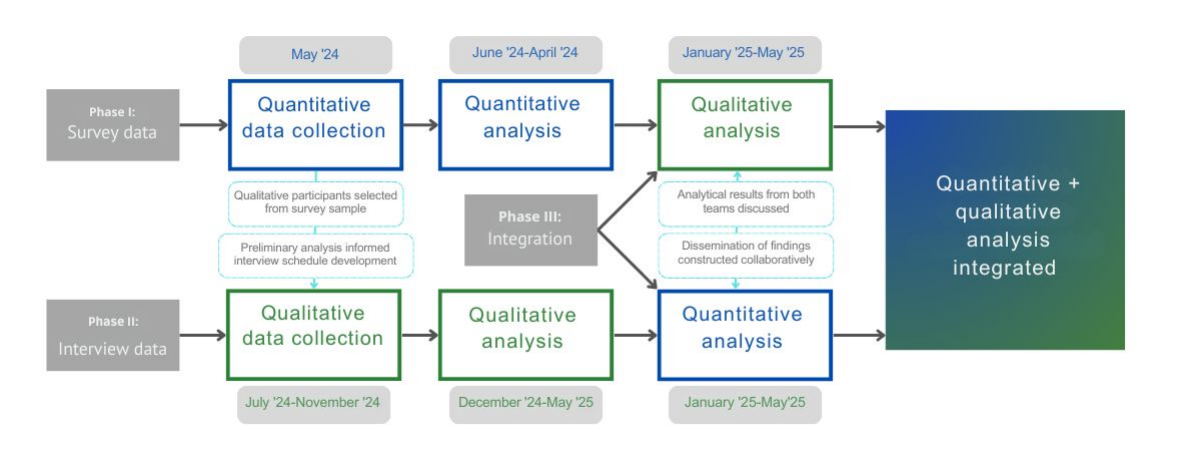
Timeline showing the points of integration between the quantitative and qualitative data.

As described in Figure 3, the threads to be investigated were developed collaboratively, with input from both the quantitative and qualitative teams. They were then followed in the data and analyzed collectively to ensure consensus and confirmability.

### Ethical Considerations

This study, approved by the University of Saskatchewan Research Ethics Board (BEH-4565), follows the Tri-Council Policy Statement on Ethical Conduct for Research Involving Humans. Any protocol changes require Research Ethics Board approval through amendments. Participants gave informed consent for both the quantitative and qualitative phases of this study—for the quantitative survey, they were required to express their consent (either “yes” or “no”) via a query that underlined the voluntary nature of this study, and for the qualitative phase, participants gave informed, verbal consent to be interviewed after being read a consent form that outlined the procedures, policies, and nature of the research project. Participation was entirely voluntary. Raw quantitative data were deidentified before analysis and stored on a secure server accessible only to the research team. All interview transcripts were deidentified before analysis and were stored on a secure server accessible only to the research team. Participants in the quantitative survey were a part of the Leger panel and received points for completing the survey, which could then be collected and redeemed for gift cards. The 1000 points are equivalent to approximately 1 CAD, and participants received 2000 points for their participation in completing the survey. Participants in the qualitative phase received a CAD $25 (US $18) honorarium for their participation in the interview.

## Results

### Study Progress

Both quantitative and qualitative data collection were completed in mid-2024. Quantitative analyses of survey data and qualitative interviews were transcribed, coded, and analyzed in early 2025. Mixed methods integration was completed in mid-2025. Following data analysis, the project advanced to dissemination and knowledge translation. Outputs include 3 quantitative papers (ready for publication), 1 mixed methods paper (submitted and under review), 1 qualitative paper (published) and a protocol paper (submitted and under review). A scoping review paper and a comprehensive project report are in preparation and scheduled for completion by the end of 2025. In addition, a public-facing website has been developed and launched. Study findings have been presented at various national conferences. Further knowledge-translation outputs are being finalized in 2025 to extend the reach of findings.

### Survey Participants

**Figure 4:**
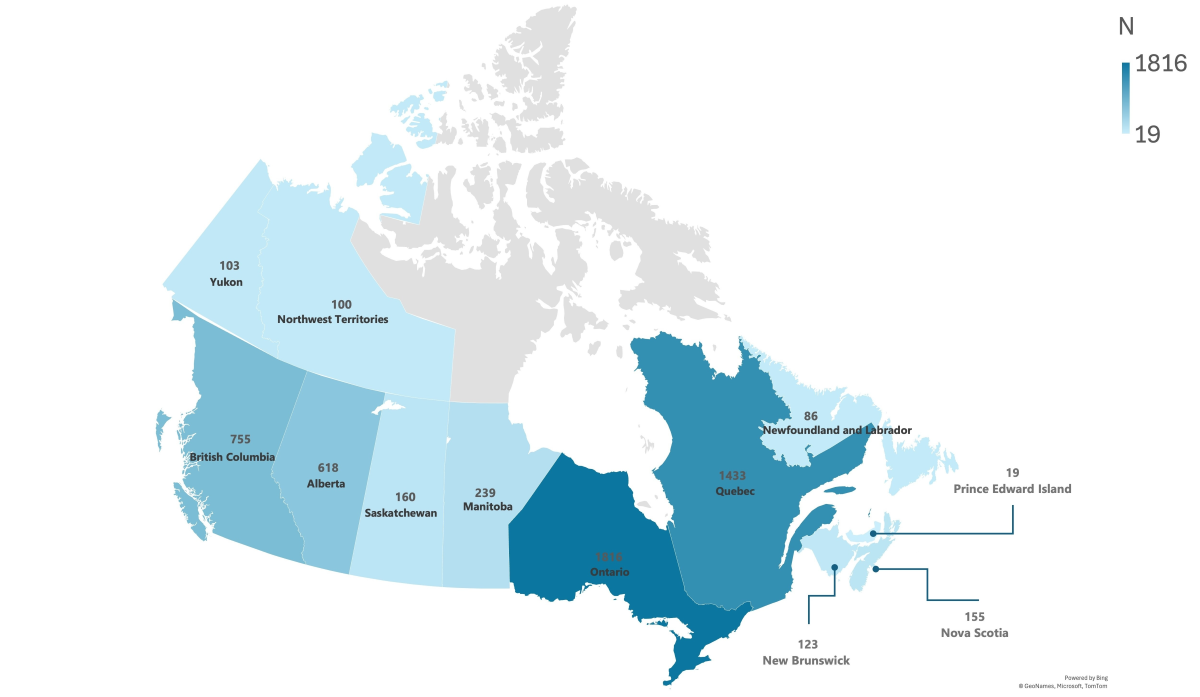
Geographic distribution of study participants by province and territory, Canada; Trust Dynamics and Equity in Public Health Project.

As described in Figure 4, the survey included participants from across Canada, with 32.4% (1816/5607) from Ontario and 25.6% (1433/5607) from Quebec, followed by 13.5% (755/5607) from British Columbia and 11% (618/5607) from Alberta. The Prairie provinces accounted for 2.9% (160/5607) from Saskatchewan and 4.3% (239/5607) from Manitoba, while the Atlantic provinces had 2.2% (123/5607) from New Brunswick, 2.8% (155/5607) from Nova Scotia, 0.3% (19/5607) from Prince Edward Island, and 1.5% (86/5607) from Newfoundland and Labrador. Additionally, 1.8% (100/5607) of participants were from the Northwest Territories and 1.8% (103/5607) from Yukon. With respect to sex assigned at birth, 45.5% (2552/5607) of participants identified as male and 54.2% (3038/5607) as female. Concerning self-reported gender identity, 45.6% (2557/5607) identified as men, 53.6% (3003/5607) identified as women, 0.52% (29/5607) identified as nonbinary, and 0.32% (18/5607) opted not to disclose their gender. Age distribution varied, with 8.1% (454/5607) aged 18-24 years, 19.3% (1080/5607) aged 25-34 years, 16.8% (942/5607) aged 35-44 years, 17% (955/5607) aged 45-54 years, 18.7% (1049/5607) aged 55-64 years, and 20.1% (1127/5607) aged 65 years or older. Additionally, 18.6% (1040/5607) identified as belonging to an ethnic minority, and 12.7% (710/5607) identified as Indigenous, including 7.2% (403/5607) First Nations, 4.8% (270/5607) Métis, and 0.4% (25/5607) Inuit. Educational attainment varied, with 0.7% (39/5607) having completed primary school or less, 19.5% (1094/5607) finishing high school, 9.2% (515/5607) attending trade school, and 69.5% (3905/5607) earning a college or university degree.

The percent trust in provincial or territorial government, federal government, public health, medical care providers, and health scientists was calculated for the whole sample (n=5607 Canadian adults). These questions specifically were asked about each group: How much did you trust the government (federal and provincial) to take care of the population during the COVID-19 pandemic? How much did you trust Public Health Authorities in their management of the COVID-19 pandemic? Did you trust the work of scientists? Responses were categorized into trust, distrust, neutral, and prefer not to answer.

Regarding COVID-19 vaccination, 55.2% (3094/5607) had received at least 1 dose and planned to stay up to date with booster recommendations, while 36.6% (2052/5607) were vaccinated but did not intend to receive additional doses. A small percentage, 0.7% (39/5607), had not yet been vaccinated but were open to it, whereas 6.4% (359/5607) had not received a vaccine and did not plan to take one.

### Interview Participants

We conducted interviews with a subset of individuals (n=41) who indicated interest in being recontacted after completing the initial quantitative survey; however, their original survey answers were not connected to their responses in the qualitative portion, preserving respondent anonymity. Initially, 101 individuals were identified by our survey partner, Leger, who had indicated they wanted to be followed up with and included either an email or phone number where they could be contacted. All 101 participants were sent a follow-up email with a link to a survey asking them for basic demographic data, as well as their availability for an interview. We received 61 responses to this survey, and all of whom were contacted to schedule an interview. Of those contacted, 41 responded and completed their interview. These participants ranged in age between 18-24 years (n=1), 25-32 years (n=7), 35-44 years (n=6), 45-54 years (n=7), 55-64 years (n=12), and 65+ years (n=8), and came from 10 different provinces and territories (Ontario, Alberta, British Columbia, Newfoundland and Labrador, Manitoba, Saskatchewan, Quebec, Nova Scotia, New Brunswick, Prince Edward Island, and Yukon).

## Discussion

Five years since the emergence of the SARS‑CoV‑2 and the COVID-19 pandemic, the world has changed irrevocably. We have experienced mass disruptions to our way of life, lost countless lives, and gained respect for how microorganisms can disrupt our lives, as well as how human ingenuity can counter these threats (by way of novel inventions such as specific vaccines and tests). Through this process, though, we have also seen an erosion of public trust—both worldwide and in Canada [22,29,44]. In Canada, public trust in governments, in particular, was called into question [29]. Canadians indicated that they did not feel the government was being consistent or transparent in its communication—frequent changes in public health messaging only served to bolster this mistrust [29,44]. Further, research suggests that lower institutional trust mirrored vaccine trust, with individuals who did not trust government or public health messaging less likely to accept a vaccine [45]. For instance, individuals who did not trust government warnings about the risk posed by COVID-19 to their communities were shown to be significantly less likely to be vaccinated [46]. This, combined with the proliferation of misinformation that the Canadian government was not fully equipped to address, resulted in many individuals feeling reluctant to take a vaccine [47]. Further, the loss of trust that occurred in both governments and scientists during the pandemic represents a major, ongoing challenge for Canadian public health.

More worldwide, a recent 23-country survey highlights that challenges of trust and vaccine hesitancy extend well beyond Canada. Conducted in October 2023 with 23,000 participants (1000 per country), collectively representing nearly 60% of the world’s population, the study defined vaccine acceptance as having received at least one COVID-19 vaccine dose and hesitancy as not having received any [48]. The results showed a marked decline in intent to receive a COVID-19 booster—from 87.9% in 2022 to 71.6% in 2023—indicating growing pandemic and vaccine fatigue. At the same time, 60.8% of respondents expressed greater willingness to receive vaccines for diseases other than COVID-19, while 23.1% reported being less willing. Trust in vaccine information sources was modest overall, with doctors or nurses (6.9/10) rated highest, while social media and family or friends ranked among the least trusted (typically <5/10). Canada reflected this broader pattern, with health care professionals emerging as the most trusted information source, and social networks rated lower. These international findings reinforce our Canadian results: trust in scientific and medical expertise remains relatively resilient, while institutional and informal sources face greater skepticism. Together, they highlight the global challenge of sustaining confidence in reliable health information while countering mistrust associated with governments and social media.

The project described in this protocol comprehensively addresses this issue. This study’s methodological framework demonstrates the importance of capturing both statistical data and in-depth experiential insights to fully understand complex the social phenomena, public trust.

This study will integrate quantitative and qualitative data to facilitate a more rigorous and comprehensive analysis of trust dynamics. A systematic analysis of these data sources will enable a deeper investigation into the underlying determinants of public trust and their implications for health behaviors. Moreover, the dissemination of findings through peer-reviewed academic publications, policy briefs, and targeted community engagement initiatives will be prioritized to ensure that the insights generated inform evidence-based decision-making and contribute to the refinement of public health strategies aimed at strengthening pandemic preparedness and response.

We will compile project findings into various academic outputs, including journal papers, research posters, technical reports, conference presentations, and infographics, which will also be stored in the Saskatchewan COVID-19 Archive.

While the mixed methods design was a distinct strength, it also had limitations. Confidentiality considerations did not allow for linking of survey responses and qualitative interviews. This limitation restricted the ability to analyze individual experiences with corresponding survey outcomes. However, qualitative themes were analyzed to complement and contextualize the overall survey findings, allowing for a broader yet meaningful integration and interpretation of public trust, trust dynamics, and trustworthiness. This approach balanced data privacy considerations while still capturing the depth and complexity of public trust.

Implementation of the quantitative strand, especially self-reported data, may be subject to recall and social desirability biases. Social desirability bias occurs when respondents answer in ways they believe will be viewed favorably, rather than truthfully. This bias can lead to overreporting trust and downplaying mistrust. Although the models adjusted for many theoretically relevant variables, there is still a tendency for the presence of residual confounding because other potential confounders, such as political ideology, media habits, and conspiracy beliefs, were not directly measured.

In conclusion, the observed decline in public trust before and during the COVID-19 pandemic presents a significant challenge for policy makers, public health authorities, and scientific institutions. Rebuilding and maintaining trust necessitates a commitment to transparent communication, accountability, and the mitigation of structural inequities that perpetuate distrust. This research provides critical insights that can inform the development of resilient, equity-driven public health frameworks, ensuring that future public health crises are met with greater public confidence and compliance.

## Appendix

Multimedia Appendix 1 Survey questionnaire.

Multimedia Appendix 2 Interview topic guide.

## Abbreviations

DGTL: digital
G10: Group of Ten
LEO: Leger Opinion

## Data Availability

Data collected in this work are archived for preservation and freely accessible online [49]. The datasets generated or analyzed during this study are available from the corresponding author on reasonable request.
