# Supplementary material for: Trust Dynamics and Equity in Public Health in Canada: Protocol for a Mixed Methods Project in the Postpandemic Era
Source: JMIR Res Protoc. 2025 Nov 28;14:e75199. doi: 10.2196/75199 (PMC12701344; doi:10.2196/75199)
Supplement: Multimedia Appendix 1 [file resprot_v14i1e75199_app1.pdf]

Trust Dynamics and Equity in Public Health:
A Mixed-Methods Study

Quota: Total sample (n=5,600)

-4600 gen pop representative sample of the Canadian population the focuses on age, gender, ethnicity (weighting will be used) – Leger DGTL for n=200 completed surveys with people living in the NWT and Yukon (100 in each)

-500 Indigenous Canadians

-500 18-34 Canadians

-n=1,400 Canadians who are considered Lower Vaccine Uptake (see below) (a minimum of 25% of total sample). may fall out slightly higher or lower but all best efforts to reach a minimum of 25% will

	SHOW CONSENT TO EVERYONE.	1. I consent to participate in this research in accordance with the conditions described above:<br>a. Yes<br>b. No

Screening Questions

SCR	Were you residing in Canada during COVID-19 pandemic and up until July 1, 2023? Please select the option that best describes your situation	○ Yes, I was in Canada for most or all of the pandemic and until July 1, 2023<br>○ No, I was not in Canada for most or all of the pandemic period [TERMINATE]<br>○ I arrived in Canada after July 1, 2023 [TERMINATE]
SCR2	Which of the following best describes you? Please select one response only<br><br>[B,C,D,E – MARK AS LOW VACCINE UPTAKE]	○ I have received at least one dose of a COVID-19 vaccine and plan to stay up to date on additional booster recommendations<br>○ I have received at least one dose of a COVID-19 vaccine and do not plan on receiving any additional COVID 19 vaccinations.<br>○ I have not received a COVID-19 vaccine but may in the future.<br>○ I have not received a COVID-19 vaccine and do not plan to in the future<br>○ Don't know/ Prefer not to answer
E1	What province do you live?	
E2	What city do you live in?	
E3	What type of area do you live?<br><br>Choose one only.	○ Rural/Country Area<br>○ Suburban/Regional<br>○ Urban/City<br>○ I don't know/ I prefer not to answer

E4	How long have you been in Canada?<br><br>Choose one only.	○ I was born in Canada<br>○ 5 years and more<br>○ Less than 5 years<br>○ I prefer not to answer
E4a	What is your sex at birth?<br><br>Sex at birth: typically assigned at birth based on a person's reproductive system and other physical characteristics.	○ Male<br>○ Female<br>○ I prefer not to answer
E5	What is your <u>gender</u>?<br><br>Gender: an individual's personal and social identity as a man, woman or non-binary person	○ Man<br>○ Woman<br>○ Non-binary person<br>○ I prefer not to answer
E6	What is your age?<br><br>Choose one only.	○ 18 – 24 years old<br>○ 25 – 34 years old<br>○ 35 – 44 years old<br>○ 45 – 54 years old<br>○ 55 – 64 years old<br>○ 65 years +
E7	Do you consider yourself an ethnic minority?<br>*The following groups make up most of the visible minority population: South Asians, Chinese, Blacks, Filipinos, Latin-Americans, Arab or Middleastern, Southeast Asians, West Asians, Koreans, and Japanese.*<br><br>*Please note that Indigenous people are not considered an ethnic minority.*<br><br>Choose one only.	○ Yes<br>○ No<br>○ I prefer not to answer
E8	How do you identify your race/ethnicity?<br><br>Select all that apply.	○ African/Black (including African-American, African-Canadian, Caribbean)<br>○ East Asian (e.g., Chinese, Taiwanese, Japanese, Korean, etc.)<br>○ European/White<br>○ Indo-Caribbean, Indo-African, Indo-Fijian, West-Indian<br>○ Latin, South or Central American<br>○ Polynesian (e.g., Samoans, Tongan, Niuean, Cook Island Maori, Tahitian Maaohi, Hawaiian Ma'oli, Marquesan, New Zealand Maori)<br>○ South Asian (e.g., Afghan, Nepali, Tamil, Bangladeshi, Pakistani, Indian, Sri Lankan, Punjabi)

		○ Southeast Asian (e.g., Vietnamese, Thai, Cambodian, Malaysian, Filipino/a, Laotian, Singaporean, Indonesian)<br>○ West Asian (e.g., Iraqi, Jordanian, Palestinian, Saudi, Syrian, Yemeni, Armenian, Iranian, Israeli, Turkish)<br>○ Prefer to self-identify: ___________<br>○ I prefer not to answer
E9	Do you identify yourself as Indigenous?<br><br>Choose one only.	○ Yes<br>○ No<br>○ I prefer not to answer
E10	If yes, please self- identify:<br>[IF YES AT E9]	○ First Nations (status/non-status)<br>○ Métis<br>○ Inuit<br>○ Prefer to self-identify: ________

QFILT1

A. Trust in Government

In this part of the survey, we're asking about your feelings of **trust in the government** (provincial and federal) and how they may have changed **before and during** the COVID-19 pandemic.

		**Provincial Government**	**Federal Government**
A1	How much did you trust the government to **take care of the population during the COVID-19 pandemic?**[1]<br><br>Choose one only.	○ Strongly Trust<br>○ Trust<br>○ Neutral<br>○ Distrust<br>○ Strongly Distrust<br>○ I prefer not to answer	○ Strongly Trust<br>○ Trust<br>○ Neutral<br>○ Distrust<br>○ Strongly Distrust<br>○ I prefer not to answer
PROGRAMMER NOTE: KEEP QUESTION ORDER AS IS, I.E., PROVINCIAL QUESTIONS TO BE ASKED BEFORE FEDERAL		The upcoming questions will cover your trust in the **provincial government before and during** the COVID-19 pandemic. Please focus on the bold and underlined words.	The following questions will cover your trust in the **federal government before and during** the COVID-19 pandemic. Please focus on the bold and underlined words.
A2	**Before the COVID-19 pandemic**, to what extent did the following factors influence your level of trust in **the provincial/ federal government?**	1 (Very Low) ,2, 3, 4, 5,6,7 (Very High)<br><br>○ Transparency and accountability (Meaning, Communication Clarity & Responsibility)<br>○ Consistency of messaging (Keeping the government statements the same)<br>○ Personal experiences<br>○ Media coverage (TV & Online news)	1 (Very Low) ,2, 3, 4, 5. 6,7 (Very High)<br><br>○ Transparency and accountability (Meaning, Communication Clarity & Responsibility)<br>○ Consistency of messaging<br>○ Personal experiences

	Likert Chart.	○ Political influences	○ Media coverage (TV & Online news)<br>○ Political influences
A2a:	**ASK THIS ON NEXT PAGE INSTEAD OF 'OTHER' WITH GRID WITH A2**	Did any other factors influence your trust in <u>the provincial government before COVID-19 pandemic</u>?<br>○ Yes, please specify<br>○ No	Did any other factors influence your trust in <u>the federal government before the COVID-19 pandemic</u>?<br>○ Yes, please specify<br>○ No
A3	**<u>During the COVID-19 pandemic</u>**, to what extent did the following factors influence your level of trust in **<u>the provincial/ federal government?</u>**<br><br>Likert Chart	1 (Very Low) ,2, 3, 4, 5,6,7 (Very High)<br>○ Transparency and accountability (Meaning, Communication Clarity & Responsibility)<br>○ Consistency of messaging (Keeping the government statements the same)<br>○ Personal experiences<br>○ Media coverage (TV & Online news)<br>○ Political influences	1 (Very Low) ,2, 3, 4, 5, 6,7 (Very High)<br>○ Transparency and accountability (Meaning, Communication Clarity & Responsibility)<br>○ Consistency of messaging (Keeping the government statements the same)<br>○ Personal experiences<br>○ Media coverage (TV & Online news)<br>○ Political influences
A3a	[PROGRAMMER NOTE: ASK THIS ON NEXT PAGE, INSTEAD OF 'OTHER' AT A3]	Did any other factors influence your trust in <u>the provincial government during the COVID-19 pandemic</u>?<br>○ Yes, please specify<br>○ No	Did any other factors influence your trust in <u>the federal government during the COVID-19 pandemic</u>?<br>○ Yes, please specify<br>○ No
A4	How much did you trust the government in terms of **<u>telling you the truth</u>** about the COVID-19 pandemic**?**<br><br>Choose one only.	○ Always<br>○ Mostly<br>○ Sometimes<br>○ Almost never<br>○ Never<br>○ I prefer not to answer	○ Always<br>○ Mostly<br>○ Sometimes<br>○ Almost never<br>○ Never<br>○ I prefer not to answer
A5	Did you trust the government officials to **<u>make the right decision</u>** during the COVID-19 pandemic?[3]	○ Yes<br>○ No<br>○ I prefer not to answer	○ Yes<br>○ No<br>○ I prefer not to answer

	Choose one only.		
A6	Please provide your level of agreement for the following statement: **<u>During the COVID-19 pandemic, the government did everything necessary to stop the virus from spreading.</u>**<br><br>Choose one only.	○ Strongly disagree<br>○ Somewhat disagree<br>○ Neither agree nor disagree<br>○ Somewhat agree<br>○ Strongly agree<br>○ I prefer not to answer	○ Strongly disagree<br>○ Somewhat disagree<br>○ Neither agree nor disagree<br>○ Somewhat agree<br>○ Strongly agree<br>○ I prefer not to answer
A7	Please provide your level of agreement for the following statement: **<u>During the COVID-19 pandemic, the government communicated with citizens effectively.</u>**<br><br>Choose one only.	○ Strongly disagree<br>○ Somewhat disagree<br>○ Neither agree nor disagree<br>○ Somewhat agree<br>○ Strongly agree<br>○ I prefer not to answer	○ Strongly disagree<br>○ Somewhat disagree<br>○ Neither agree nor disagree<br>○ Somewhat agree<br>○ Strongly agree<br>○ I prefer not to answer
A8	Please provide your level of agreement for the following statement: **<u>The government has sufficient expertise to lead the country/ province.</u>**<br><br>Choose one only.	○ Strongly disagree<br>○ Somewhat disagree<br>○ Neither agree nor disagree<br>○ Somewhat agree<br>○ Strongly agree<br>○ I prefer not to answer	○ Strongly disagree<br>○ Somewhat disagree<br>○ Neither agree nor disagree<br>○ Somewhat agree<br>○ Strongly agree<br>○ I prefer not to answer

B. Trust in Health Authorities

The following survey section will be about trust in **<u>Public Health Authorities (</u>**Public Health Agency of Canada (PHAC), and Provincial and Territorial Health Authorities (e.g. Ontario Health, Toronto Public Health, Vancouver Coastal Health, Nova Scotia Health Authority, Health PEI, Saskatchewan Health Authority)

	Matrix	**Strongly Trust**	**Trust**	**Neither Trust nor**	**Distrust**	**Strongly Distrust**	I prefer not to answer

				**Distrust**			
B1	How much did you trust Public Health Authorities' **management of the COVID-19 pandemic?** [4]						
B2	How much did you trust the **sources of information** provided by Public Health Authorities during the pandemic? [4]						
B3	How much did you trust the **decision-making processes and strategies** employed by Public Health Authorities in responding to pandemic or dealing with COVID-19? [4]						
B4	How much trust did you place in the **effectiveness and transparency of communication** from Public Health Authorities regarding the COVID-19 situation? [4]						
B5	How much trust did you place in the **prevention efforts** (eg. masking, social distancing **EXCLUDING** vaccination) from Public Health Authorities regarding the COVID-19 situation?						
B6	How much trust did you place in the **vaccination efforts** from Public Health Authorities regarding the COVID-19 situation?						
B7	How confident are you in Public Health Authorities' ability to handle **unexpected health emergencies in the future?** [4]	Very Confident	Somewhat Confident	Neutral	Not very Confident	Not at all confident	

		Increased Trust	Remained the Same	Decreased Trust	I prefer not to answer
B8	How has your trust in Public Health Authorities changed since the beginning of the COVID-19 pandemic?[5]<br><br>Choose one only.				

The following section will be about trust in **health scientists and science[7-8]**

In this part of the survey, we're asking about your agreement of trust in science and scientists in the **context of the COVID-19 pandemic.**

	Question	Answer
B9	I trust the **work** of scientists [7-8]<br><br>Choose one only.	○ Strongly disagree<br>○ Somewhat disagree<br>○ Neither agree nor disagree<br>○ Somewhat agree<br>○ Strongly agree
B10	I trust that **scientists** are being honest in their work [7-8]<br><br>Choose one only.	○ Strongly disagree<br>○ Somewhat disagree<br>○ Neither agree nor disagree<br>○ Somewhat agree<br>○ Strongly agree
B11	I trust that scientists are being **ethical** in their work [7-8]<br><br>Choose one only.	○ Strongly disagree<br>○ Somewhat disagree<br>○ Neither agree nor disagree<br>○ Somewhat agree<br>○ Strongly agree
B12	People who understand science well have more **trust** in science [7-8]<br><br>Choose one only.	○ Strongly disagree<br>○ Somewhat disagree<br>○ Neither agree nor disagree<br>○ Somewhat agree<br>○ Strongly agree
B13	I cannot trust the **COVID-19 vaccine** because it was developed too quickly [7-8]<br><br>Choose one only.	○ Strongly disagree<br>○ Somewhat disagree<br>○ Neither agree nor disagree<br>○ Somewhat agree<br>○ Strongly agree

QFIL2

B14. Has your trust in **<u>Health Scientists/ Science</u>** changed since the beginning of the COVID-19 pandemic? Choose one only.

- Increased Trust
- Remained the Same
- Decreased Trust
- I prefer not to answer.

Please tell me how strong your interest is in each of the following topics.

	Matrix	Very Strong	Somewhat Strong	Neutral	Somewhat Low	Low	Not at all
B15	Politics						
B16	Science and Research						
B17	Local or National News						
B18	Economics and Finance						
B19	Religious or Spiritual beliefs						

B20. Please provide your level of agreement for the following statement:

<u>Political decisions should be based on scientific evidence.</u> Choose one only.

- Strongly disagree
- Somewhat disagree
- Neither agree nor disagree
- Somewhat agree
- Strongly agree
- I prefer not to answer

The following survey section will be about trust in **<u>medical care providers or doctors</u>**.

	Matrix	**Strongly Trust**	**Trust**	**Neutral**	**Distrust**	**Strongly Distrust**
B21	**<u>Before COVID-19</u>** (before March 2020), how much did you trust that your doctor could provide **<u>safe and effective healthcare</u>**?[5]					
B22	**During COVID-19,** how much did you trust that your doctor could provide **<u>safe and effective healthcare</u>**?					

C. Trust in Social and Community Networks

These questions are about trust in **family members, friends, organizations and media** that you encounter in your daily life.

C1 Matrix		How **often** did you use the following sources to get information about **public health updates during COVID-19 pandemic?**[6]					
	**Information Sources**	**Never**	**Rarely**	**Occasionally**	**Frequently**	**Always**	**I prefer not to answer**
C1a	Federal Government (Health Canada, Federal Minister of Health)						
C1b	Provincial Government						
C1c	Public Health Authorities (Public Health Agency of Canada, Public Health Units)						
C1d	Newspapers and other print media						
C1e	Radio, podcasts and other broadcasts						
C1f	Television News						
C1g	Family or Friends						
C1h	Healthcare Provider (i.e. Nurses, Pharmacists, Doctors)						
C1i	Celebrities or Social Media Influencers						
C1j	Social Media Platforms (i.e. Facebook, X (Twitter), YouTube)						
C1k	Religious or Faith Leaders						

C2. How much did you **trust** the following sources to get information about public health updates **during COVID-19 pandemic?**[6]						
**Information Sources** Matrix	**Strongly Trust**	**Trust**	**Neither Trust or Distrust**	**Distrust**	**Strongly Distrust**	**I prefer not to answer**
C2a. Federal Government (i.e. Briefings, Website)						
C2b. Provincial Government (i.e. Briefings, Website)						
C2c. Newspapers and other print media (e.g. blogs, newsletters)						
C2d. Radio, podcasts and other broadcasts						

C2e. Public Health Authorities (i.e. Health Canada, Public Health Agency of Canada, Public Health Units)						
C2f. Television News						
C2g. Family or Friends						
C2h. Healthcare Provider (i.e. Nurses, Pharmacists, Doctors)						
C2i. Celebrities or Social Media Influencers						
C2j. Social Media Platforms (i.e. Facebook, Twitter, YouTube)						
C2k. Religious or Faith Leaders						

D. Interpersonal Trust

This section has questions about your own **trust and belief about COVID-19 vaccines**.

	Question	Answer
D1	Indicate your level of trust in COVID-19 vaccines?[4]<br><br>Choose one only.	○ Strongly trust<br>○ Trust<br>○ Neutral<br>○ Distrust<br>○ Strongly distrust
D2	From your personal perspective, how willing were you to receive COVID-19 vaccination?[4]<br>Choose one only.	○ Very unwilling<br>○ Somewhat unwilling<br>○ Neutral<br>○ Somewhat willing<br>○ Very willing<br>○ I prefer not to answer
D3	What type of COVID-19 vaccine(s) have you received? Select all that apply.	❑ Moderna (Spikevax)<br>❑ Pfizer/BioNTech (Comirnaty)<br>❑ AstraZeneca Vaxzevria<br>❑ Janssen Jcovden (Johnson & Johnson)<br>❑ Other (please specify)<br>❑ I have been vaccinated but do not know the vaccine type<br>❑ I did not receive COVID-19 vaccine<br>○ I prefer not to answer
D4	I did not get COVID-19 vaccine, because:[4]<br>[THOSE WHO DID NOT RECEIVE THE VACCINE AT SCR2 – C,D,E]<br>Select all that apply	○ I do not have trust in vaccines in general<br>○ I do not trust COVID-19 vaccine<br>○ I do not trust the COVID-19 vaccine approval process<br>○ Not enough testing has been done<br>○ I did not think COVID-19 is a threat<br>○ I have concern about safety<br>○ Other (Please specify)_____________________________<br>○ I prefer not to answer [exclusive]
D5	I am not sure about getting the vaccine because:[4.]<br>[THOSE WHO DID NOT RECEIVE THE VACCINE AT SCR2 – C,D,E]	○ I want to see more scientific evidence<br>○ I do not think I need one<br>○ I want to wait and see other's experience with them<br>○ I have concern about safety<br>○ Other (Please specify)_____________________________

	Select all that apply	○ I prefer not to answer[exclusive]
D6	Have you **ever refused a vaccine** that was recommended to you by a healthcare professional?<br>Choose one only.	○ Yes<br>○ No<br>○ I prefer not to answer
D7	What was the **reason**(s) for refusing a vaccine recommended by a healthcare professional? [YES AT D6]<br>Select all that apply.	❑ I did not have enough information on the vaccine<br>❑ I was concerned about side effects<br>❑ I did not think the vaccine was effective<br>❑ I had a bad experience with a previous vaccination<br>❑ I did not know where to get vaccination<br>❑ Other (please specify)<br>❑ I prefer not to answer
D8	Do you think that your family members should be vaccinated against COVID-19, regardless of their vaccination status?<br>Choose one only.	○ Definitely should be<br>○ Probably should be<br>○ Maybe<br>○ Probably should not be<br>○ Definitely should not be<br>○ I prefer not to answer
(D8A - ASK AFTE R D8)	Have any of your family members received the COVID-19 vaccine?<br>Choose one only.	○ Yes<br>○ No<br>○ I prefer not to answer
D9	Has **social media** (Facebook, X (Twitter), Instagram, Snapchat, etc.) influenced your perceptions towards receiving the COVID-19 vaccination?<br>Choose one only.	○ Yes<br>○ No<br>○ I prefer not to answer
D10	Please specify the **social media source(s)** that had influence.<br><br>[YES AT D9] Select all that apply.	○ Facebook<br>○ X (Twitter)<br>○ Instagram<br>○ Snapchat<br>○ TikTok<br>○ Other (specify)_____<br>○ I prefer not to answer [exclusive]
D11	Has **official media** (news channels, radio stations, podcasts, newspapers, etc.) influenced your perceptions towards receiving the COVID-19 vaccination?<br>Choose one only.	○ Yes<br>○ No<br>○ I prefer not to answer
D12	Please specify **the official media source(s)** that had influence.	○ News Channels (e.g. CBC News, CTV News, Global News)<br>○ Radio Stations (e.g. CBC Radio One, 680 News, local radio stations)

	[YES AT D11] Select all that apply.	○ Podcasts<br>○ Newspapers (e.g. Globe and Mail, local newspapers)<br>○ Other (specify)______<br>○ I prefer not to answer [exclusive]
D13	What **sources** influenced your decision to get or not to get an initial COVID-19 vaccination?<br>Select all that apply.	❑ Healthcare professionals<br>❑ Government officials<br>❑ Friends and family<br>❑ Social media (Facebook, X (Twitter), Instagram, Snapchat, etc.)<br>❑ Official media (News channel, radio stations, newspapers, etc.)<br>❑ Other (please specify)<br>❑ I prefer not to answer (Ex

The following questions are for **demographic purposes** only.

E. Demographics

E11	On a scale from 1 to 7, with 1 being 'Not religious at all' and 7 being 'Extremely religious,' how religious would you consider yourself to be?	Not religious at all 1,2,3,4,5,6,7 Extremely religious
E12	Do you adhere to spiritual practices or beliefs?<br>Choose one only.	• Yes<br>• No<br>• I prefer not to answer
E13	What is your highest level of **completed education**?<br>Choose one only.	○ Primary/elementary school or less<br>○ Secondary/high school<br>○ Trade School<br>○ College or University degree<br>○ I have never been to school<br>○ I prefer not to answer
E14	What is your **occupation** sector?<br>Select all that apply.	○ Business, Finance and Administration<br>○ Engineering, Architecture, Computer and Information Technology<br>○ Healthcare, Science and Research<br>○ Education, law and social, community and government services<br>○ Art, culture, recreation and sport<br>○ Sales and Service Occupations<br>○ Trades, Transportation and Equipment Operations<br>○ Natural Resources, Agriculture and Production<br>○ Manufacturing and Utilities<br>○ Not Employed (Unemployed, Student, Retired)<br>○ I prefer not to answer

E15	What is your marital status?<br><br>Choose one only.	○ Single (never married)<br>○ Married, or living together<br>○ Widowed<br>○ Divorced<br>○ Separated<br>○ I prefer not to answer
E16	What is your **parental status**?<br>Select all that apply	○ Not a parent [EXCLUSIVE]<br>○ Parent of at least one child under 10 years old<br>○ Parent of at least one child aged 11-17<br>○ Parent of at least one child aged 18 and older<br>○ I prefer not to answer[EXCLUSIVE]
E17	Please indicate how many people aged **17 and below** you currently live with you?<br>Choose one only.	○ None<br>○ 1 person<br>○ 2 people<br>○ 3 people<br>○ 4 or more people<br>○ I prefer not to answer
E18	Please indicate how many people aged **18-64** currently live with you?<br>Choose one only.	○ None<br>○ 1 person<br>○ 2 people<br>○ 3 people<br>○ 4 or more people<br>○ I prefer not to answer
E19	Please indicate how many people aged **65 or older** currently live with you?<br>Choose one only.	○ None<br>○ 1 person<br>○ 2 people<br>○ 3 people<br>○ 4 or more people<br>○ I prefer not to answer
E20	Please select a category that matches your **annual household income** before tax?<br>Choose one only.	○ Under $15,000<br>○ $15,000 - $24,999<br>○ $25,000 - $34,999<br>○ $35,000 - $44,999<br>○ $45,000 - $59,999<br>○ $60,000 - $69,999<br>○ $70,000 - $79,999<br>○ $80,000 - $89,999<br>○ $90,000 - $99,999<br>○ $100,000 or over<br>○ I prefer not to answer
E21	What is your **employment status**?<br>Choose one only.	○ Employed full-time<br>○ Employed part-time<br>○ Self-employed<br>○ Unemployed (actively seeking employment)<br>○ Unemployed (not actively seeking employment)<br>○ Student (full-time)<br>○ Student (part-time)

		○ Retired<br>○ Homemaker<br>○ Disabled, unable to work<br>○ I prefer not to answer<br>○ Other (please specify)

Would you be willing to be contacted for the follow-up interview during the second part of the study? If you would like to share more of your experience and are willing to participate in a 30-minute interview, please provide your contact information below. **Your personal information will not be associated with any of your responses to this survey.** It will not be shared and will only be used for the purpose of contacting you to discuss your experiences. If you choose to participate and if you are selected, we will compensate you for your time. Please provide either a phone number or an email address as a required form of contact.

Name: ____

Telephone: ____________________________________

Email: ____

☐ Not Interested/ Prefer not to answer

Thank you for your participation!
