# Supplementary material for: Trust Dynamics and Equity in Public Health in Canada: Protocol for a Mixed Methods Project in the Postpandemic Era
Source: JMIR Res Protoc. 2025 Nov 28;14:e75199. doi: 10.2196/75199 (PMC12701344; doi:10.2196/75199)
Supplement: Multimedia Appendix 2 [file resprot_v14i1e75199_app2.pdf]

Dear participant,

We are reaching out to you because you recently participated in a survey on trust issues during the pandemic in Canada. You indicated that you would be interested in being contacted for a follow-up interview. Thank you again for being willing to contribute to this study. Your participation is a crucial part of helping us to understand how trust played out during the pandemic. We are really looking forward to talking with you.

To help us get to know a little bit more about you, we ask that you take a moment to answer the quick survey below. Your answers will help us prepare the interview questions as well as learn a bit more about you. These questions might seem familiar from the questionnaire you already participated in – to ensure anonymity, your survey answers stay separate from any data you provide to us during the interview process, so we need to access some of that basic demographic information for a second time. The survey also has questions about your availability so we can set up a time for an interview.

https://www.surveymonkey.ca/r/LTT525L

We plan to schedule interviews throughout July and August that can last anywhere between fifteen minutes to an hour. The interview style will be conversational. If there is something you are interested in speaking about, we encourage you to do so! The interview is a completely non-judgemental space to tell us your story.

After you respond to this survey, a member of our team will be in touch to schedule an interview and provide some more details surrounding ethics, consent, and things for you to consider as you prepare.

Thank you again for being a part of this important work.

Kind regards,

Fionnuala Braun
Research Assistant
Coronavirus Variants Rapid Response Network (CoVaRR-Net)
University of Saskatchewan Historical GIS Lab
fib085@usask.ca

CoVaRR-Net HGIS LAB

Dear Participant,

Thank you for agreeing to take part in the qualitative interview portion of **Trust Dynamics and Equity in Public Health: A Mixed-Methods Study on COVID-19.** This study engages with topics of health and trust over the entire period of COVID-19, and also acknowledges that your trust in certain organizations may have shifted as the pandemic went on. Therefore, our research team has put together a small guide to help you reflect on how your trust in public institutions was affected during the pandemic.

The purpose of this guide is to help you organize your thoughts by providing a general overview of the topics that may be discussed during your interview. It will also help you to remember different parts of the pandemic, and engage with how your feelings shifted as the pandemic has gone on.

Before your interview…

1. Think back to the first few months of the pandemic (March 2020-August 2020), and how you made decisions about the health and safety of yourself and your family members during this period. What was important to you? How did you keep yourself and others safe?
2. Reflect on how you understand the difference between **government** and **scientists.** Try to remember if you perceived these groups differently during the pandemic, and if you trusted one more than the other.
3. Reflect on how you understand the difference between **governments** (federal, provincial, or municipal), **health authorities**, and **community leaders and family.** Recall how you trusted or did not trust these different groups as the pandemic progressed.
4. Think if there is one group (governments, health authorities, or community leaders and family) that particularly affected your trust during the pandemic, or that you would just be interested in talking about more.
5. What are some lessons about trust that you learned during the pandemic? What are some ideas to improve trust that you would like to pass on to policymakers as we better learn to deal with COVID-19?

Once again, our research team thanks you for taking the time to both prepare for and participate in this study. Your insights provide invaluable information that will be used to improve the quality of public healthcare across the country.

Please feel free to reach out to a member of our research team if you have any questions.

Best wishes,

Trust Dynamics and Equity Research Team

UNIVERSITY OF SASKATCHEWAN

Appendix 1 – Qualitative Interview Guide

**Topics**[1]	
Introduction	**Brief researcher introduction, including your name and your position on the TRUST project.** I really appreciate you taking the time to talk with me a little bit today about your experience with trust during the pandemic.
Aim of the study	The aim of this study is to learn more about trust in health professionals, government authorities, and scientists throughout the pandemic, and how these levels of trust might have fluctuated. We're looking to provide a comprehensive set of results that can be used to inform public health policy and help us prepare for future public health crises.
Information/consent form	You should have received a consent for and a package detailing the questions and purpose of the research. **Read through consent form and obtain verbal consent.** Do you have any questions about this?
Withdrawal, anonymity, and confidentiality	You can tell me at any time during the interview that you no longer wish to participate. If there are any questions you feel uncomfortable answering, please feel free to tell me this as well. I will be recording our conversation, and this data will be stored confidentially (further details needed about anonymity and confidentiality here).
Documenting experiences	This study is not about passing judgement on personal beliefs – it's about learning how healthcare providers and governments can serve everyone better. There are no right or wrong answers, and absolutely no judgement. Talking about issues surrounding trust, particularly about a highly politicized topic like vaccines can be sensitive, so feel free to take your time.
Recording	Do you have any questions before we get started? If not, I'll start recording.

Interview

**COVID-19 Initial Experiences**<br>o Introduction<br>o Initial months of the pandemic<br>o Questions and concerns that began to arise<br>o What sources were most frequently accessed for information
To begin, I would like to get to know a little bit more about you, and your experiences during the pandemic. This will help me to better understand the answers you give during the later parts of the interview, and let us talk a bit before we get into some of the issues surrounding trust.<br>• Tell me a bit more about those initial months of COVID-19. When did you realize that this was going to be something lifechanging?<br>• What were the main places you looked for information about COVID-19 during 2020 (government, media, social media, community leaders)?[2]<br>• Why did you choose to trust the information put out by these sources?

[1] Adapted from Simon, Barbara, Marij Hillen, Johanna Aarts, Jacqueline Tromp, Eline de Heus & Saskia Duijts. "Disentangling trust of patients with rare cancer in their healthcare professionals and the healthcare system: A qualitative interview study." *Journal of Cancer Survivorship* (2024).

[2] Adapted from Shiroma, Kristina, Tara Zimmerman, Bo Xie, Kenneth R. Fleischmann, Kate Rich, Min Kyung Lee, Nitin Verma, & Chenyan Jia. "Older Adults' Trust and Distrust in COVID-19 Public Health Information: Qualitative Critical Incident Study." *JMIR Aging* 6 (2023).

**Defining Trust**
Now, I would like to talk a little bit more about trust during the pandemic. This section will focus on how you define trust, and what trust looks like in your life.<br>• What does trust mean to you? (By this I mean are you someone who quickly trusts someone/something, or not?)[3]<br>• What are some key things that help you determine whether a person or organization is trustworthy?

**Trust in Public Health Measures/Vaccines**
Now that we've spoken a bit about what trust means to you, I would like you to keep that definition in mind while I ask you about some of the public health interventions we experienced in Canada during the pandemic.<br>• What do you think governments and public health actors **were trying to achieve** when they introduced pre-vaccine public health measures (by this I mean masking, social distancing, shutting down businesses)?<br>• What do you think scientists and researchers **were trying to achieve** in their development of a COVID-19 vaccine?<br>• Do you feel that scientists and researchers were transparent about COVID-19 vaccine development? Tell me more about this.<br>• If you could go back to when vaccines were first introduced, would you choose to take a vaccine? Is this a different decision than what you originally chose?

**Fluctuating Trust in Public Information**
That last question provided a transition into the next part of this interview, which is going to look a little bit at whether your trust in different information sources changed over the course of the pandemic. When discussing this, I'm going to ask you to reflect on changes in opinion you might have experienced. Don't worry if you can't remember exactly how you felt – the closest approximation is completely okay. Mostly, I'd like to hear more from you what caused any potential changes in your levels of trust.<br>• Did your trust in **government information** (federal, provincial, or municipal politicians) change from the beginning of the pandemic to where we are now? If so, why?<br>• Did your trust in **health authorities** (public health agencies, doctors, scientists) change from the beginning of the pandemic to where we are now? If so, why?<br>• Did your trust in **community leaders and family** (faith leaders, friends, family) change from the beginning of the pandemic to where we are now? If so, why?<br>Previous research indicates that, in situations where trust is observed as fluid, there are certain dimensions that influence levels of trust.[4] I'd like to hear a little bit more about how some dynamics

[3] Simon et al. "Disentangling trust of patients with rare cancer in their healthcare professionals and the healthcare system," 2024.

[4] Dasch, Selina, Jonas Wachinger, Till Bärnighausen, Simiao Chen and Shannon A. McMahon. "Deliberation, context, emotion and trust – understanding the dynamics of adults' COVID-19 vaccination decisions in Germany." *BMC Public Health* 23, no. 136 (2023); Straten, G.F.M., R.D. Friele, and P.P. Groenewegen. "Public trust in Dutch health care." *Social Science & Medicine* 55, no. 2 (2002); Baker, David W. "Trust in Health Care in the Time of

influenced your trust in _____ (pick the institution above that the person said the most about, or the one they seemed the most invested in discussing).

- Can you tell me a bit about your experience with **communication** (between experts and citizens, between various expert groups, experts and policymakers) in relation to _____ during the pandemic?
- Can you tell me about your experience with **beneficence** (belief that experts have citizens' best interests at heart) in relation to ____ during the pandemic?
- Can you tell me about **competence** (belief that experts have the necessary expertise to recommend the best course of actions for citizens) in relation to ____ during the pandemic?
- Can you tell me about **honesty** (extent to which participants believes that government/experts/community was honest and provided reliable and up-to-date information) in relation to ____ during the pandemic?
- Of the dynamics of trust indicated (communication, beneficence, competence, honesty), which one affected your trust in _____ the most? Why?

Moving Forward

Coming to the end of the interview, I'd like to know a little bit more about what you think could have been done differently, either by the government, experts, or your immediate community during the pandemic.

- Imagine there is another pandemic in the future. What advice would you give to decisionmakers or scientists that you think would help citizens to trust them more?

References

Baker, David W. 2020. "Trust in Health Care in the Time of COVID-19." *JAMA* 324, no. 20.

Dasch, Selina, Jonas Wachinger, Till Bärnighausen, Simiao Chen and Shannon A. McMahon. 2023. "Deliberation, context, emotion and trust – understanding the dynamics of adults' COVID-19 vaccination decisions in Germany." *BMC Public Health* 23, no. 136.

Shiroma, Kristina, Tara Zimmerman, Bo Xie, Kenneth R. Fleischmann, Kate Rich, Min Kyung Lee, Nitin Verma, & Chenyan Jia. 2023. "Older Adults' Trust and Distrust in COVID-19 Public Health Information: Qualitative Critical Incident Study." *JMIR Aging* 6.

Simon, Barbara, Marij Hillen, Johanna Aarts, Jacqueline Tromp, Eline de Heus & Saskia Duijts. 2024. "Disentangling trust of patients with rare cancer in their healthcare professionals and the healthcare system: A qualitative interview study." *Journal of Cancer Survivorship*.

Straten, G.F.M., R.D. Friele, and P.P. Groenewegen. 2002. "Public trust in Dutch health care." *Social Science & Medicine* 55, no. 2.

Tanny, Tahmina Ferdous. 2022. "Dynamics of Trust: A Review of Trust in Government." *Indian Journal of Public Administration* 69, no. 1.

COVID-19." *JAMA* 324, no. 20 (2020); Tanny, Tahmina Ferdous. "Dynamics of Trust: A Review of Trust in Government." *Indian Journal of Public Administration* 69, no. 1 (2022).
